# Large-bodied birds are over-represented in unstructured citizen science data

**DOI:** 10.1038/s41598-021-98584-7

**Published:** 2021-09-24

**Authors:** Corey T. Callaghan, Alistair G. B. Poore, Max Hofmann, Christopher J. Roberts, Henrique M. Pereira

**Affiliations:** 1 German Centre for Integrative Biodiversity Research (iDiv) Halle-Jena-Leipzig, Puschstr. 4, 04103 Leipzig, Germany; 2 Ecology and Evolution Research Centre, School of Biological, Earth and Environmental Sciences, UNSW Sydney, Sydney, NSW Australia; 3 Institute of Biology, Martin Luther University Halle-Wittenberg, Am Kirchtor 1, 06108 Halle (Saale), Germany

**Keywords:** Ecology, Biodiversity

## Abstract

Citizen science platforms are quickly accumulating hundreds of millions of biodiversity observations around the world annually. Quantifying and correcting for the biases in citizen science datasets remains an important first step before these data are used to address ecological questions and monitor biodiversity. One source of potential bias among datasets is the difference between those citizen science programs that have unstructured protocols and those that have semi-structured or structured protocols for submitting observations. To quantify biases in an unstructured citizen science platform, we contrasted bird observations from the unstructured iNaturalist platform with that from a semi-structured citizen science platform—eBird—for the continental United States. We tested whether four traits of species (body size, commonness, flock size, and color) predicted if a species was under- or over-represented in the unstructured dataset compared with the semi-structured dataset. We found strong evidence that large-bodied birds were over-represented in the unstructured citizen science dataset; moderate evidence that common species were over-represented in the unstructured dataset; strong evidence that species in large groups were over-represented; and no evidence that colorful species were over-represented in unstructured citizen science data. Our results suggest that biases exist in unstructured citizen science data when compared with semi-structured data, likely as a result of the detectability of a species and the inherent recording process. Importantly, in programs like iNaturalist the detectability process is two-fold—first, an individual organism needs to be detected, and second, it needs to be photographed, which is likely easier for many large-bodied species. Our results indicate that caution is warranted when using unstructured citizen science data in ecological modelling, and highlight body size as a fundamental trait that can be used as a covariate for modelling opportunistic species occurrence records, representing the detectability or identifiability in unstructured citizen science datasets. Future research in this space should continue to focus on quantifying and documenting biases in citizen science data, and expand our research by including structured citizen science data to understand how biases differ among unstructured, semi-structured, and structured citizen science platforms.

## Introduction

Citizen science, or community science,—the involvement of volunteers in scientific endeavors—is increasingly seen as a cost-effective method for biodiversity monitoring and research. Accordingly, the quantity and diversity of citizen science projects in the ecological and environmental sciences is increasing^1^. Such projects are quickly accumulating hundreds of millions of biodiversity observations around the world annually^2,3^ expanding the spatial and temporal scope of our understanding in ecology, conservation, and natural resource management^4,5^. Citizen science projects vary widely in their scope, design, and intent^6–8^. Projects can range from unstructured (e.g., little training needed to participate and contribute opportunistic/incidental observations) to semi-structured (e.g., with minimal workflows and guidelines, but additional data collected with each observation can be included) to structured (e.g., prescribed sampling in space and time by mostly trained and experienced volunteers). The level of structure consequently influences the overall data quality of a particular project^9,10^.

Data quality from citizen science projects has been questioned^11,12^, and such concerns can act as a barrier to the widespread use of citizen science data in ecology and conservation^13^. These concerns arise because citizen science data can be biased temporally, spatially, and/or taxonomically. Temporally, many citizen science datasets are biased because participants frequently sample on weekends^14^ or disproportionately during specific times of the year such as spring migration for birds^15^, or during specific times of day, such as the morning period when birds are most active. Spatially, there is often a disproportionate number of sightings from areas with large human populations^16^, areas with more accessibility^17^, regions with high biodiversity that attract observers^18^, and regions of the world with higher socioeconomic status^19^. Taxonomic biases also exist as some taxa receive orders of magnitude more citizen science observations than other taxa, evidenced by the fact that birds represent a disproportionate amount of data in the Global Biodiversity Information Facility^2^. Even within citizen science projects focused on specific taxa, there can be strong taxonomic biases towards particularly charismatic species or those that are readily identified^20–23^.

Despite potential biases in citizen science datasets, contrasts of data from unstructured projects to those contributed by more structured projects have shown that citizen science programs can provide reliable data^12,24^. For example, one case study found that mark-recapture models of whale sharks are reliable whether using sightings reported by the public or by experienced researchers^25^, and another case study found that unstructured data performs comparably with structured data in identifying and monitoring invasive plant species^26^. When analyzed appropriately, citizen science data can further our understanding of many facets of biodiversity, including estimating species distributions^27–29^, managing habitat for conservation^30^, estimating species richness^31^, monitoring pollination services^32^, and quantifying population trends^33,34^. In such examples, statistical solutions to account for known biases and noise inherent in citizen science data are used^3,35,36^.

In addition to being an excellent resource for scientists to better understand ecological questions, citizen science projects can encourage increased engagement of the general public with science^37,38^. Many citizen science projects provide learning opportunities for their volunteers. For example, participants in citizen science projects have increased their knowledge about invasive weeds^39–41^, increased their knowledge of bird biology and behavior^42^, and even enhanced their conservation awareness and sense of place^42,43^. The ecological advances derived from citizen science data, combined with the important role it plays in community engagement with science, suggests that citizen science data will continue to play an important role in ecological and conservation research in the future^2,4,38,44^. However, what motivates volunteers to participate in science, and contribute observations, has important implications for the quality of the data obtained^45^, particularly if there are biases towards certain species, places, or times of sampling.

To ensure the continued and expanded use of citizen science data in ecology and conservation, it is important to document and understand the different biases present in citizen science datasets. Importantly, the degree of bias in a particular dataset will be influenced by the level of structure of that citizen science project. For example, unstructured projects (e.g., iNaturalist, www.inaturalist.org) or semi-structured projects (e.g., eBird, www.ebird.org) will generally be more spatially biased than structured projects that have pre-defined spatial sampling locations (e.g., Breeding Bird Surveys). Or, a citizen science project that collects incidental presence-only data, such as iNaturalist, is likely more susceptible to individual observer preferences compared with a semi-structured or structured project that requires all species encountered to be recorded by the observers. Charismatic species^21^ can be over-represented in citizen science data because observers are more likely to record species that they, or society, consider more interesting or relevant for monitoring^46^. Similarly, rare species are more likely to be the subject of conservation monitoring or more likely to be actively searched for by amateur naturalists^47,48^ and thus can be over-represented in biodiversity datasets. In contrast, in some citizen science projects, abundant species can form a disproportionate number of records (e.g.,^49^) because species’ abundance can lead to an increase in the number of records by casual observers^50^. Differences in species detectability^50^, and the ease of making the observations, also lead to taxonomic biases in citizen science datasets. Therefore, species traits (e.g., body size, color, flock size) may have an additive effect, influencing both the detectability of a species^51–53^, and in turn, the likelihood of a species being submitted to an unstructured citizen science database.

**Figure 1:**
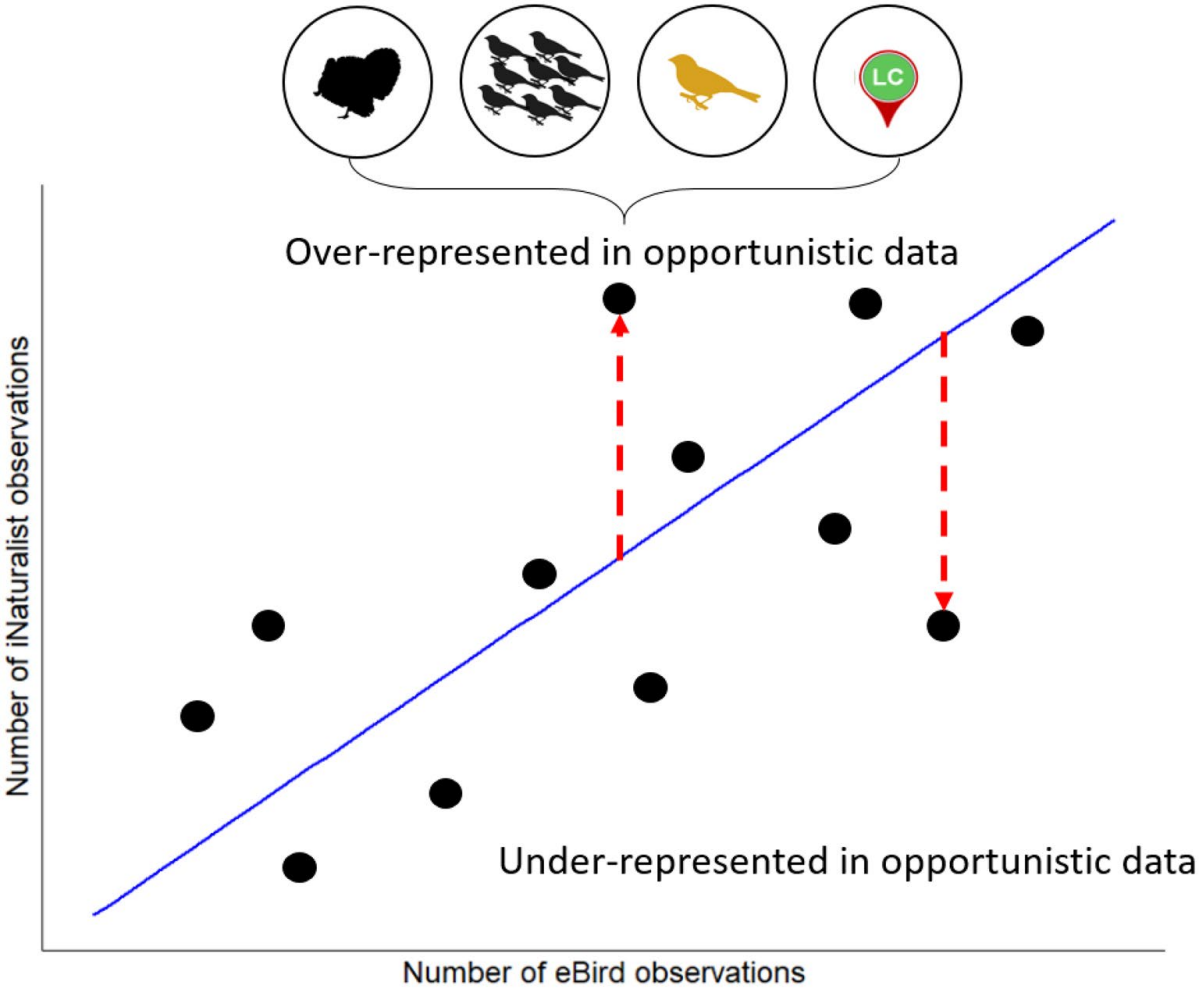
A conceptual figure depicting the methods used in our analysis. We used the residual from the relationship between the number of log10-transformed eBird observations (i.e., semi-structured citizen science observations) and log10-transformed iNaturalist observations (i.e., unstructured citizen science observations) to quantify the over- or under-representation of a species in unstructured citizen science data. We predicted that species which were over-represented in unstructured iNaturalist data would be larger in size, occur more frequently in large flocks, be brighter in color, and be categorized as Least Concern IUCN status (a proxy for commonness).

Quantifying biases in citizen science datasets can help (1) researchers using these data to better account for biases when drawing ecological conclusions, (2) the design and implementation of future citizen science projects, and (3) understand what species or regions may need data collection from professional scientists by understanding the ‘limits’ of citizen science projects^19^. Here, we quantify biases in bird observation data from an unstructured, citizen science project—iNaturalist—with that from a semi-structured one—eBird. We restricted our comparison to birds because (1) birds are among the most popular taxa with the non-scientific public, ensuring large sample sizes in both citizen science projects, and (2) data on the species traits that may influence the likelihood of unstructured observations are readily available for birds. We assessed the over-representation or under-representation of bird species’ observations in the unstructured citizen science project compared to the semi-structured project (see Fig. 1). We then tested the following predictions: that (1) more colorful species; (2) larger species; (3) species with the ‘least concern’ IUCN status; and (4) more gregarious species (i.e., with larger flock sizes) are over-represented in the unstructured citizen science dataset (iNaturalist) in contrast to the semi-structured citizen science dataset (eBird). Our analysis highlights the importance of considering species’ traits when using citizen science data in ecological research.

## Methods

We made comparisons between iNaturalist (www.inaturalist.org)—an unstructured citizen science project—and eBird (www.ebird.org)—a semi-structured citizen science project^15,54^.

*iNaturalist citizen science data.* iNaturalist is a multi-taxon citizen science project hosted by the California Academy of Sciences. It is an unstructured citizen science project where volunteers contribute opportunistic photos or sound recordings through a smart-phone or web-portal. Photos are then identified to the lowest possible taxonomic resolution using a community identification process, and once two users, or more than two-thirds, confirm the species-level identification of an organism it is considered “research grade”. Observations that are research grade are then uploaded to the Global Biodiversity Information Facility. We downloaded iNaturalist observations from the Global Biodiversity Information Facility for the contiguous United States^55^ for the period from January 2010 to May 2019, on December 3rd, 2019. For more details on the iNaturalist methodology, see here: https://www.inaturalist.org/pages/getting+started.

*eBird citizen science data.* eBird is one of the most successful citizen science projects in the world, with > 1 billion bird observations globally. It was launched in 2002 by the Cornell Lab of Ornithology and focuses on collecting reliable data on the distributions and relative abundance of birds throughout the world^54^. It is a semi-structured project where volunteers submit ‘checklists’ of birds seen and/or heard on birding outings, following different protocols (e.g., stationary, incidental, or travelling). An important component of eBird that differentiates it from unstructured data collection is that users are required to indicate whether the checklist is ‘complete’—meaning they included all species they were able to identify during that birding outing. When using complete checklists only in an analysis, a user can infer non-detections in the dataset for any species not recorded. Observers can submit checklists at any time and place of their choosing, and for any duration and distance travelled. All complete checklists additionally include the duration and distance travelled while birding. Filters are set—based on spatiotemporal coordinates—which restrict the species and their associated counts that can be added to the downloadable eBird dataset without approval from a regional expert reviewer^56^. We used the eBird basic dataset (version ebd_May-2019) and restricted our analysis to data from the contiguous United States for the period from January 2010 to May 2019. We also restricted the data used to those of the best ‘quality’ by excluding incomplete checklists, checklists that were incidental or historical, which travelled > 5 km, lasted < 5 min, and lasted > 240 min, minimizing the leverage of outliers on analyses^57,58^.

*Filtering and aggregating the citizen science datasets*. Although both datasets are global in scope, we restricted our analysis to the contiguous United States as both of these citizen science projects initiated in the United States, and thus the data are most numerous from there. For comparisons, we aggregated data at the state level. This was done to account for differences that may exist throughout the entirety of the United States including differences in user behavior and the species pools that differ geographically. We used the eBird Clements taxonomy (version 2018) and all species from iNaturalist were matched with this taxonomy. A total of 1030 species was initially collated from the eBird checklists, but many of these only occurred once or a few times—possibly representing misidentifications that had not yet been fixed by local reviewers or escaped and exotic birds which are incorporated in the eBird dataset but not considered part of the local avifauna or of interest to our analysis here. Although, these could represent scarce and uncommon species in a state as well, albeit these are rarely sampled by iNaturalist. To account for these biases, we removed species that were on < 1% of eBird checklists for a given state; trimming the eBird observations to the ‘core’ suite of species that occur in a state (sensu^57^). After trimming the species and harmonizing the taxonomy with iNaturalist, there were 507 species remaining which were considered in our main analyses presented throughout the results. Although our results here are presented using the 1% cutoff level, we tested the sensitivity of this cutoff level and found comparable results across 0, 0.5, 1, and 1.5% cutoffs. For each state, the eBird and iNaturalist data were summarized by calculating the total number of observations in that state for every species where an observation represents a single unique sighting of a species for both iNaturalist and eBird. Using these aggregated data, we conducted preliminary comparisons of the unstructured and semi-structured datasets by quantifying the relationship between the number of eBird checklists and iNaturalist observations at the state level, and the number of unique observations at the species level. We also explored the relationship between the proportion of checklists a species was found on and the proportion of all iNaturalist observations a species represented at the state level.

### Species-specific over- or under-representation in iNaturalist

Our first analytical step was to model the log–log linear relationship between the total number of observations in iNaturalist and total number of observations in eBird for a species (Fig. 1). This linear model was repeated separately for each state, where the response variable was log-transformed number of iNaturalist observations and the predictor variable was log-transformed number of eBird observations. We repeated the model by state to account for inherent differences among states that were not of interest in our particular analysis, such as (1) the number of observers in a state, (2) the different relative abundance of a species throughout the United States, and (3) any other intrinsic differences that might exist among states that was not of interest in our analysis. A species with a high (i.e., positive) residual would be over-represented in iNaturalist relative to eBird, whereas a species with a low (i.e., negative) residual would be under-represented in iNaturalist relative to eBird (Fig. 1). First, we visualized these state-specific residuals along the trait variables to empirical summarize and visualize the results of this methodological approach. Second, we took the residuals from these models and used these as the response variables in our subsequent analyses of species trait characteristics (see below).

### Species-specific trait data

We tested whether four predictor variables (see Fig. 1) would explain the over- or under-representation of bird species in the unstructured citizen science data. For each species, we used a proxy for their commonness/abundance, categorized according to IUCN status, taken from HBW BirdLife international checklist version 3 (http://datazone.birdlife.org/species/taxonomy). This variable was treated as an ordinal variable in our models (see below) and encompassed Least Concern, Vulnerable, and Near Threatened species. The three species recorded as endangered were removed from this analysis due to a lack of statistical power at this level with so few observations. For each species we used the continuous predictor variables of (1) body size; (2) color; and (3) average flock size. Body sizes (adult body mass in grams) were taken from the amniote life history database compiled by Myhrvold et al.^59^ and were log-transformed to meet normality assumptions. Color was taken from Dale et al.^60^ and was extracted as RGB values for six patches per species (upper breast, lower breast, crown, forehead, nape, throat). To define a continuum of color where the brightest/most colorful (and likely most detectable species based on color) had the highest value we combined both the ‘distance from brown’ and the ‘brightness’ of a species for the data from Dale et al.^60^. Distance from brown was defined as the maximum Euclidian distance in the cubic RGB color space from brown (R = 102, B = 68, G = 0) from any of the six patches on a species, regardless of sex (i.e., the highest value across both sexes). Brightness was defined as the maximum relative luminance (i.e., 0.2126R + 0.7152G + 0.0722B) from any of the six patches on a species, regardless of sex. These two variables were combined and scaled from 0 to 1 for all species in Dale et al.^60^ and this value was used as our measure of color. Calculations were done in “Lab” space, an approximately perceptually uniform color space standardized by the Commission Internationale d'Eclairage. Exploratory analyses showed similar results with HSV color space. Flock size—an approximation of the gregariousness of a species—was taken from eBird as the average number of reported individuals among all checklists where a species was reported, across all data. We acknowledge that the number of a species reported on an eBird checklist likely encompasses both the gregariousness of a species as well as the density of a species in an area, as birders can travel through multiple territories. However, qualitative exploration of the flock size variable aligned with a priori expectations of average flock size (Table S1).

### Statistical analysis

We used mixed effects models to examine the effects of species traits on the relative bias between our unstructured and semi-structured citizen science datasets. The response variable was the residuals from a log–log linear model fit between the eBird observations and the iNaturalist observations for a given species (described above), the predictor variables were the respective traits, and the random effect (i.e., random intercept) was state. By using state as a random effect, we accounted for any replication of some species across states and the varying degree of over, or under-representation of that species across states. First, we ran a global model where all traits were included as predictor variables: log10-transformed body size, log10-transformed flock size, color, and IUCN status treated as an ordinal variable where Least Concern was coded as the highest variable and Near Threatened as the lowest. Second, to confirm the results of this global model, we ran four separate models—one for each trait as listed above—because there was much missing data for species’ traits. This approach allowed us to test the relationship of a predictor given the other predictor variables (i.e., all predictors against the response variable simultaneously) as well as the independent relationships (i.e., each predictor separately against the response variable).

### Data analysis and availability

All analyses were carried out in R software^61^ and relied heavily on the tidyverse workflow^62^. Mixed-effects models were fitted using the lme4 package^63^ and p-values were computed using the lmerTest package^64^. Data and code to reproduce these analyses are available here: 10.5281/zenodo.5509770.

## Results

**Figure 2:**
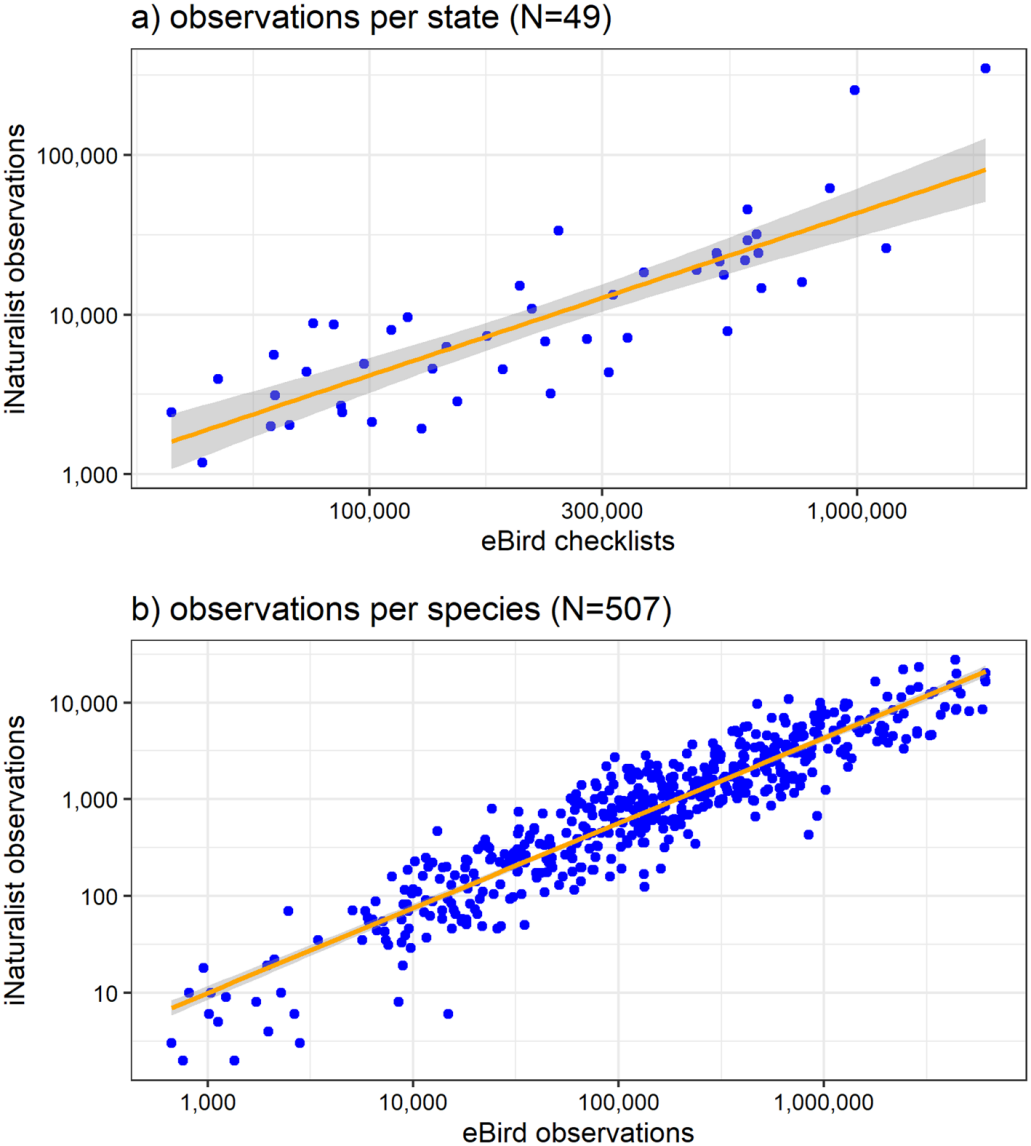
(**a**) The relationship between the total number of eBird checklists and total number of iNaturalist observations for 49 states, including the District of Columbia. There was strong evidence that these variables were correlated (R^2^ = 0.58, p-value < 0.001) suggesting that sampling between datasets is correlated among states. (**b**) The relationship between the number of observations for a species from eBird (x-axis) and the number of observations for a species from iNaturalist (y-axis) for only eBird species which were found on > 1% of eBird checklists.

A total of 255,727,592 eBird and 1,107,224 iNaturalist observations were used in our analysis. At the state level, the number of eBird checklists and the number of iNaturalist observations were strongly correlated (Fig. 2a; R^2^ = 0.58, p-value < 0.001). Similarly, at the species level, the total number of iNaturalist observations and eBird observations for a given species was strongly correlated (Fig. 2b; R^2^ = 0.9), and for both datasets the number of observations per species was positively-skewed (Figure S1). We also found that the percent of eBird checklists a species was found on and the percent of total iNaturalist observations a species comprised was strongly correlated among states (Figure S2), suggesting that species are sampled to a proportionally similar extent in unstructured and semi-structured citizen science projects.

**Figure 3:**
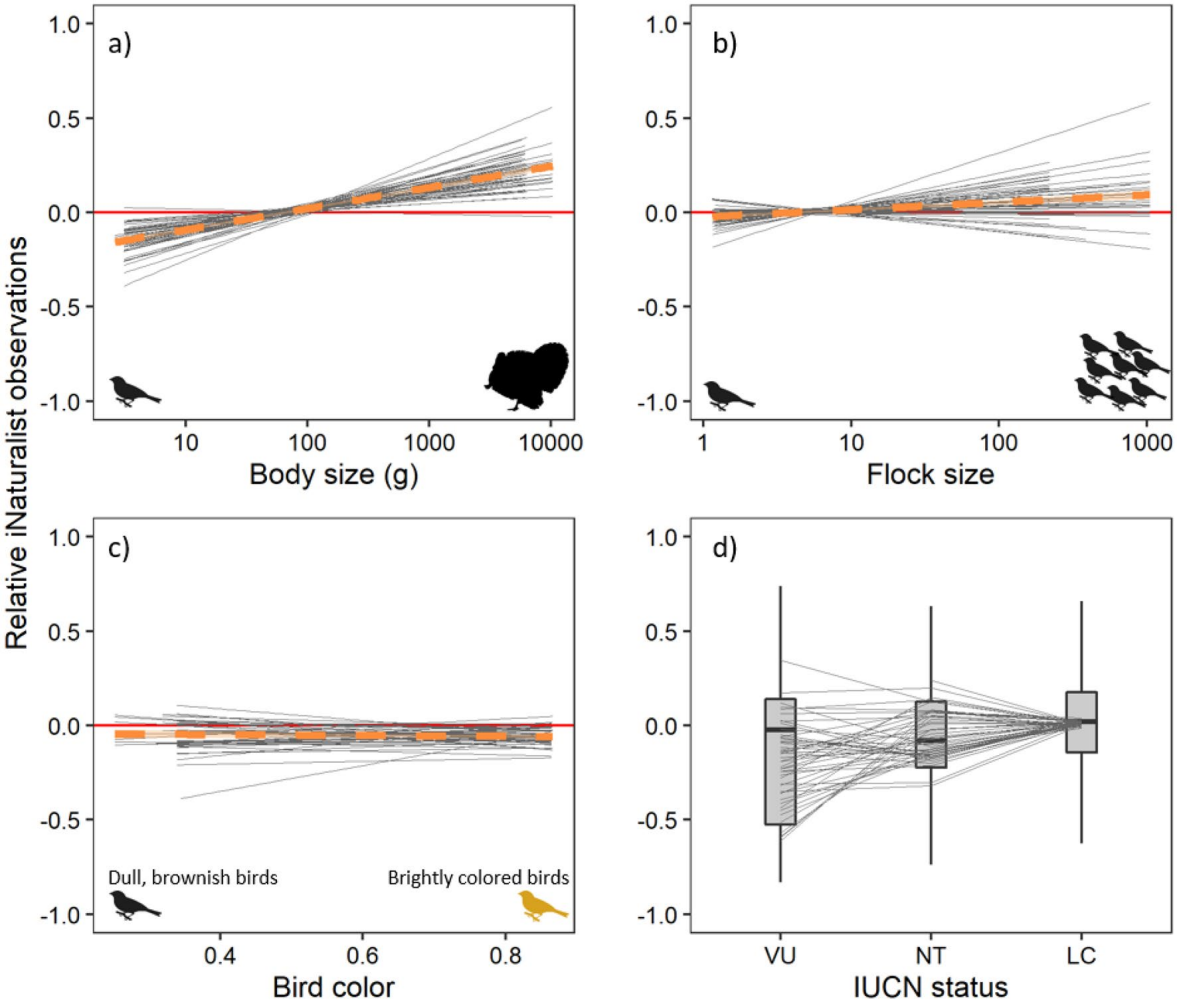
The relationship between (**a**) body size of a species, (**b**) flock size, (**c**) color and (**d**) commonness and the residuals of a linear model fit between iNaturalist and eBird observations (see Fig. 1). These empirical results demonstrate that there is a strong bias of body size in iNaturalist compared with eBird. Positive values on the y-axis mean over-represented in iNaturalist and negative values on the y-axis mean under-represented in iNaturalist. Body size and flock size are represented on a log10 scale. Each line represents a state (N = 49). For (**a**)–(**c**), the overall relationship pooling states is represented by the orange fitted line and 95% confidence interval. The data represented here were then used in our mixed effects model (see Fig. 4 for results).

Across the 507 species included in our analyses (Table S1), we showed that larger species were more likely to be over-represented in the unstructured citizen science dataset, and this was true in most states, as illustrated by the empirical comparison (Fig. 3a). The empirical comparison also showed over-representation of flock size in the unstructured dataset, although some states showed a negative relationship indicating the possibility that this trait varies in space (Fig. 3b). There was no discernible pattern in the relationship between color and over- or under-representation in iNaturalist data (Fig. 3c), and there was some evidence that Least Concern species were over-represented in the iNaturalist data (Fig. 3d).

**Figure 4:**
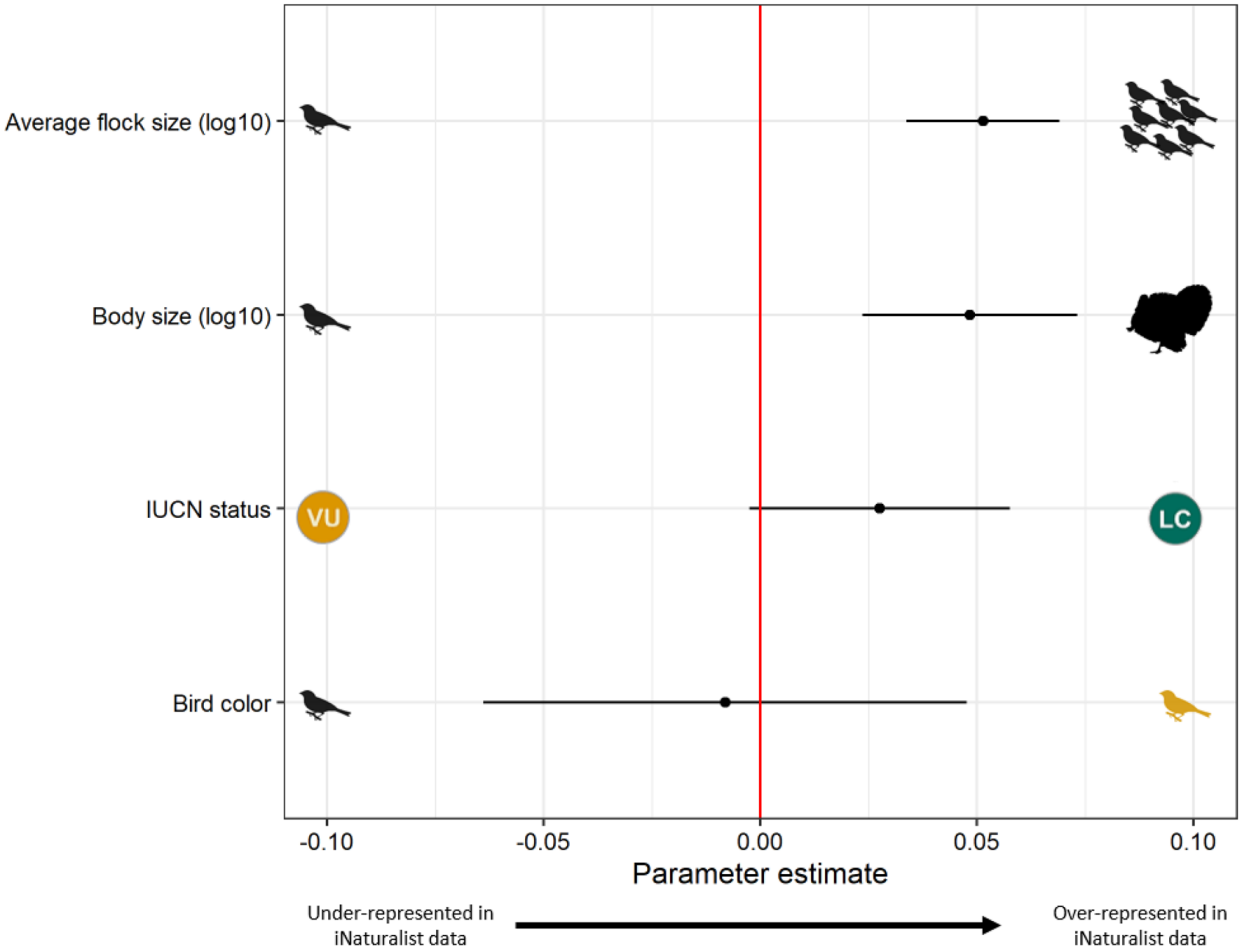
Results of a linear mixed effect model where all four variables were considered simultaneously, and state was a random effect. Strong support was found for body size and flock size (their 95% confidence interval does not overlap 0), whereas moderate support was found for IUCN status, and no support was found for color.

Using the data visualized in our empirical comparisons (Fig. 3), our mixed effects multiple regression linear model (N = 3986 observations and 222 species) with state as a random effect (Fig. 4) found strong evidence that body size (parameter estimate = 0.049; 95% CI = 0.023, 0.073) and flock size (parameter estimate = 0.051; 95% CI = 0.034, 0.069) were over-represented in iNaturalist compared with eBird; moderate evidence that common species were over-represented (parameter estimate = 0.027; 95% CI =  −0.003, 0.058); and no evidence that color influenced the over- or under-representation of a species in iNaturalist (parameter estimate =  −0.008; 95% CI =  −0.064, 0.048). The patterns found in the multiple regression model qualitatively matched that of the individual trait models, where more observations were included in some instances (see Table S2).

## Discussion

We compared two popular citizen science platforms throughout the continental United States and found that there was strong agreement between the relative number of observations of a species in iNaturalist and eBird, albeit there were about 200 times more observations in eBird than iNaturalist. This suggests that species are observed at similar rates in both citizen science projects—i.e., the inherent processes driving observation in both unstructured and semi-structured citizen science projects are similar. Nevertheless, in support of our predictions (Fig. 1) we found strong evidence that large-bodied birds are over-represented in the unstructured citizen science dataset compared with the semi-structured dataset. We also found moderate evidence that common species were over-represented in the unstructured data, and strong evidence that species in large flocks were over-represented. In contrast to our prediction, however, we found no evidence that brightly-colored species were over-represented in unstructured citizen science data.

Our finding that large-bodied birds were over-represented in an unstructured citizen science dataset, supported by our empirical comparison and mixed effects model, is probably because larger-bodied birds are more detectable (i.e., more easily found and identified)^53,65^. This confirms previous research which has shown that smaller-bodied taxa are under-represented in citizen science data^66–68^, but this may not be the case for some taxa such as mammals^69^. However, it is difficult to know whether this is an inherent preference shown by users of the unstructured citizen science data, or if this comes about as part of the recording process (e.g., species’ detectability;^50^). Species detectability is complex and can be linked to species’ mobility or habitat preferences of the species themselves; for example, large-bodied wading birds generally occurring in open wetlands are more easily detected than small-bodied songbirds generally occurring in dense forest understory.

Related to detectability, an important distinction between iNaturalist and eBird is how identifications are made. For an observer to make a record in iNaturalist, usually a photograph is uploaded (although sound recordings are also accepted). Because a photograph is needed, the submission process is two-fold—first, it needs to be detected, and second, it needs to be photographed, which is likely easier for many large-bodied birds. Longer lenses, often restricted to serious photographers, may be needed to photograph smaller-bodied birds whereas smartphones can usually capture a sufficient image of a larger-bodied bird. In contrast to iNaturalist, in eBird, a lot of identifications are made acoustically, and identification can sometimes also use contextual clues such as behavior or habitat of the bird—often difficult to capture in a photograph. Most traits analyzed here are related to visual encounter/identification, thus likely explaining the differences found between the unstructured iNaturalist and the semi-structured eBird data. To illustrate this difference, in New York state, the most under-represented species in iNaturalist (i.e., with the lowest residuals) are Marsh Wren, American Crow, Warbling Vireo, Least Flycatcher, Willow Flycatcher—all species that are identified largely acoustically. In contrast, the most over-represented species in iNaturalist (i.e., with the highest residuals) are House Sparrow, American Robin, Palm Warbler, Northern Mockingbird—all species that are easy to visually see and thus detect and photograph (Table S1). Therefore, the bias towards large-bodied birds in the unstructured data is probably a result of detectability and the ability to capture a photograph^53^. Photographs can also be uploaded to eBird, and a further test of this hypothesis could interrogate the species in eBird which have photographs uploaded. This process is similar in insects, for example, which are generally small, but larger insects (e.g., butterflies) are both easier to observe, photograph, and identify—making it likely that the biases we found in birds generalize to insects as well. Indeed, a study of bugs and beetles found that smaller species are typically less represented in citizen science data^68^. Importantly, because this body size bias is systematic, it may be easier to model, as we know that these data are not missing at random (e.g.,^70^) and thus body size can be included in various modelling processes when using unstructured citizen science data (e.g.,^67^).

Similar to body size, our mixed effects model found that birds which occur in larger groups (i.e., flocks) are over-represented in the unstructured dataset. This, again, may be inherently linked to the recording process, rather than a specific bias or preference of the observers themselves. This is because common birds, that occur in large flocks, are more likely to be seen and thus submitted to the unstructured citizen science project^65^. A larger flock will likely also provide more opportunities to capture a photograph than when observing a single individual, as has been illustrated in the detectability of animals from aerial surveys by professionals^71^. However, we note that our measure of flock size (i.e., average checklist count) may not truly represent the gregariousness of a species when observed by an eBirder or iNaturalist contributor. The variability among states in the empirical comparison of flock size (e.g., Fig. 3) illustrates that this overall bias is not uniform across all regions in the United States. Similarly, for body size there was some differentiation in the empirical comparison across states, although to a lesser extent than flock size. Such differences in bias between eBird and iNaturalist can be driven by many external factors such as the avifauna in the region, the number of eBird or iNaturalist users and the characteristics of those users. Future work should look to investigate what factors influence the biases we highlight here in space and/or time.

One explanation for the least concern birds being somewhat over-represented in iNaturalist, as supported by our mixed effect model and empirical comparison, is user behavior—eBird data are more likely to be derived from avid birdwatchers (e.g., those that search out uncommon birds and keep serious lists) compared with iNaturalist data which may be derived from more recreational birdwatchers that focus on common species—frequently the ones occurring in ‘backyards’, for example. The types of participants, and their motivations, of iNaturalist and eBird are therefore likely very different as has generally been shown among citizen science projects (e.g.,^72^). Participants submitting observations to eBird are likely better at identifying birds than those submitting to iNaturalist and can also rely on acoustic and contextual clues to make identifications, as discussed above. Importantly, our analysis focused on only unstructured versus semi-structured data, but future work should expand this comparison to include structured datasets (e.g., breeding bird surveys) to understand if the biases found here also exist when compared with more structured datasets. For example, there may be a skew in eBird data towards rare birds when compared to standardized surveys (e.g., breeding bird surveys) resulting from birders preferentially adding rare and uncommon species. Such a finding would further highlight the divergence in behavior between the users of iNaturalist and eBird.

The lack of signal of the colorfulness of a species in predicting over-representation in iNaturalist, found in both our empirical comparison and our mixed effect model, could suggest that iNaturalist users are not limited by ‘attractiveness/aesthetics’ but mostly by detectability, as discussed above (Fig. 4). Alternatively, the lack of a signal here could be a result of the comparison being between a semi-structured and an unstructured dataset—i.e., it is possible that both eBird and iNaturalist are skewed towards more colorful species, and a comparison with a structured dataset will help test this hypothesis. Quantifying the influence of color on detectability remains a challenge (e.g.,^73^). In contrast to our results, others have demonstrated a clear preference of ‘color’ by the general public in online google searches of birds^74^. However, the role of aesthetics, or color, by the public may be complex as illustrated by one study which found that only blue and yellow were significant in determining bird ‘beauty’^75^. In other taxa, more colorful insect species are more commonly reported^68^, as well as more patterned and morphologically interesting species. This may suggest, at least in the case of insects, that contributors are selecting subjects based on their visual aesthetics, not just their detectability. The discrepancies between our results and that of Caley et al.^68^ suggest that the influence of traits may vary between different taxa, making it important to explore these relationships for a range of organisms rather than extrapolating the results of birds, or bugs and beetles, to other groups.

While citizen science data are undoubtedly valuable for ecology and conservation^4,76,77^, there remain limits to the use of citizen science datasets^13,78^. The ability to sample remote regions, for example, will likely remain a limitation in citizen science data, and this has been well-recognized^17^. Quantifying the limits of citizen science datasets for use in ecology and conservation remains an important step for the future widespread use of citizen science data in ecology and conservation. Data-integration—where noisy citizen science data are integrated with professionally-curated datasets—will likely be increasingly important in the future use of citizen science data^79,80^. By knowing the biases present in citizen science data, experts can preferentially generate data that maximizes the integration process, for example by collecting data from remote regions. Further, professional scientists could use limited funding to target species that are likely to be under-represented in some citizen science datasets—i.e., rare, small-bodied, species.

Ultimately, citizen science data will continue to perform, at least in part, a substantial role in the future of ecology and conservation research^44^. Understanding, documenting, and quantifying the biases associated with these data remains an important first step before the widespread use of these data in answering ecological questions and biodiversity monitoring^5^. Our results highlight that for birds, semi-structured eBird has many more observations than unstructured iNaturalist data, but the number of observations recorded per species are strongly correlated between the two platforms. When looking at the differences in this relationship, it is clear that biases exist, likely due to the biases in the unstructured iNaturalist data. We note that we compared the unstructured dataset to a semi-structured dataset, and the semi-structured dataset does not necessarily represent the “truth”. The biases found here, could also be present when comparing a semi-structured dataset to true density or abundance of birds in the landscape. To better understand these differences, future research in this space should continue to focus on quantifying and documenting biases in citizen science data, and understanding how these biases change from unstructured to semi-structured to structured citizen science platforms. Nevertheless, our results demonstrate the importance of using species-specific traits when modelling citizen science data^27,29,52,81–84^.

## Supplementary Information

Supplementary Information 1.

Supplementary Table S1.

## References

[1] Pocock MJ, Tweddle JC, Savage J, Robinson LD, Roy HE (2017). The diversity and evolution of ecological and environmental citizen science. PLoS ONE.

[2] Chandler M (2017). Contribution of citizen science towards international biodiversity monitoring. Biol. Cons..

[3] Chandler, M. *et al.* Involving citizen scientists in biodiversity observation. In *The GEO Handbook on Biodiversity Observation Networks* 211–237 (Springer, 2017).

[4] McKinley DC (2017). Citizen science can improve conservation science, natural resource management, and environmental protection. Biol. Cons..

[5] Pereira, H. M. *et al.* Monitoring essential biodiversity variables at the species level. In *The GEO Handbook on Biodiversity Observation Networks* 79–105 (Springer, 2017).

[6] Wiggins, A. & Crowston, K. From conservation to crowdsourcing: A typology of citizen science. in *2011 44th Hawaii International Conference on System Sciences* 1–10 (IEEE, 2011).

[7] Haklay, M. Citizen science and volunteered geographic information: Overview and typology of participation. In *Crowdsourcing Geographic Knowledge* 105–122 (Springer, 2013).

[8] Kelling S (2019). Using semistructured surveys to improve citizen science data for monitoring biodiversity. Bioscience.

[9] Welvaert M, Caley P (2016). Citizen surveillance for environmental monitoring: Combining the efforts of citizen science and crowdsourcing in a quantitative data framework. Springerplus.

[10] Callaghan CT, Rowley JJ, Cornwell WK, Poore AG, Major RE (2019). Improving big citizen science data: Moving beyond haphazard sampling. PLoS Biol..

[11] Bonter DN, Cooper CB (2012). Data validation in citizen science: A case study from project FeederWatch. Front. Ecol. Environ..

[12] Kosmala M, Wiggins A, Swanson A, Simmons B (2016). Assessing data quality in citizen science. Front. Ecol. Environ..

[13] Burgess HK (2017). The science of citizen science: Exploring barriers to use as a primary research tool. Biol. Cons..

[14] Courter JR, Johnson RJ, Stuyck CM, Lang BA, Kaiser EW (2013). Weekend bias in citizen science data reporting: Implications for phenology studies. Int. J. Biometeorol..

[15] Sullivan BL (2014). The eBird enterprise: An integrated approach to development and application of citizen science. Biol. Cons..

[16] Kelling S (2015). Can observation skills of citizen scientists be estimated using species accumulation curves?. PLoS ONE.

[17] Tiago P, Ceia-Hasse A, Marques TA, Capinha C, Pereira HM (2017). Spatial distribution of citizen science casuistic observations for different taxonomic groups. Sci. Rep..

[18] Geldmann J (2016). What determines spatial bias in citizen science? Exploring four recording schemes with different proficiency requirements. Divers. Distrib..

[19] Callaghan CT (2021). Three frontiers for the future of biodiversity research using citizen science data. Bioscience.

[20] Ward DF (2014). Understanding sampling and taxonomic biases recorded by citizen scientists. J. Insect Conserv..

[21] Troudet J, Grandcolas P, Blin A, Vignes-Lebbe R, Legendre F (2017). Taxonomic bias in biodiversity data and societal preferences. Sci. Rep..

[22] Martín-López, B., Montes, C., Ramírez, L. & Benayas, J. What drives policy decision-making related to species conservation? *Biol. Conserv.* **142**, 1370–1380 (2009).

[23] Boakes EH (2010). Distorted views of biodiversity: Spatial and temporal bias in species occurrence data. PLoS Biol.

[24] Aceves-Bueno E (2017). The accuracy of citizen science data: A quantitative review. Bull. Ecol. Soc. Am..

[25] Davies TK, Stevens G, Meekan MG, Struve J, Rowcliffe JM (2013). Can citizen science monitor whale-shark aggregations? Investigating bias in mark–recapture modelling using identification photographs sourced from the public. Wildl. Res..

[26] Crall AW (2011). Assessing citizen science data quality: An invasive species case study. Conserv. Lett..

[27] van Strien AJ, van Swaay CA, Termaat T (2013). Opportunistic citizen science data of animal species produce reliable estimates of distribution trends if analysed with occupancy models. J. Appl. Ecol..

[28] Johnston A, Moran N, Musgrove A, Fink D, Baillie SR (2020). Estimating species distributions from spatially biased citizen science data. Ecol. Model..

[29] Tiago P, Pereira HM, Capinha C (2017). Using citizen science data to estimate climatic niches and species distributions. Basic Appl. Ecol..

[30] Sullivan BL (2017). Using open access observational data for conservation action: A case study for birds. Biol. Cons..

[31] Callaghan CT (2020). Citizen science data accurately predicts expert-derived species richness at a continental scale when sampling thresholds are met. Biodivers. Conserv..

[32] Birkin L, Goulson D (2015). Using citizen science to monitor pollination services. Ecol. Entomol..

[33] Delaney DG, Sperling CD, Adams CS, Leung B (2008). Marine invasive species: Validation of citizen science and implications for national monitoring networks. Biol. Invasions.

[34] Schultz CB, Brown LM, Pelton E, Crone EE (2017). Citizen science monitoring demonstrates dramatic declines of monarch butterflies in western north america. Biol. Cons..

[35] Bird TJ (2014). Statistical solutions for error and bias in global citizen science datasets. Biol. Cons..

[36] Isaac NJ, van Strien AJ, August TA, de Zeeuw MP, Roy DB (2014). Statistics for citizen science: Extracting signals of change from noisy ecological data. Methods Ecol. Evol..

[37] Dickinson JL (2012). The current state of citizen science as a tool for ecological research and public engagement. Front. Ecol. Environ..

[38] Bonney R (2014). Next steps for citizen science. Science.

[39] Jordan RC, Gray SA, Howe DV, Brooks WR, Ehrenfeld JG (2011). Knowledge gain and behavioral change in citizen-science programs. Conserv. Biol..

[40] Crall AW (2013). The impacts of an invasive species citizen science training program on participant attitudes, behavior, and science literacy. Public Underst. Sci..

[41] Jordan RC, Ballard HL, Phillips TB (2012). Key issues and new approaches for evaluating citizen-science learning outcomes. Front. Ecol. Environ..

[42] Evans C (2005). The neighborhood nestwatch program: Participant outcomes of a citizen-science ecological research project. Conserv. Biol..

[43] Haywood BK, Parrish JK, Dolliver J (2016). Place-based and data-rich citizen science as a precursor for conservation action. Conserv. Biol..

[44] Pocock, M. J. *et al.* A vision for global biodiversity monitoring with citizen science. In *Advances in Ecological Research* vol. 59, 169–223 (Elsevier, 2018).

[45] Tiago P, Gouveia MJ, Capinha C, Santos-Reis M, Pereira HM (2017). The influence of motivational factors on the frequency of participation in citizen science activities. Nat. Conserv..

[46] Isaac NJ, Pocock MJ (2015). Bias and information in biological records. Biol. J. Lin. Soc..

[47] Angulo E, Courchamp F (2009). Rare species are valued big time. PLoS ONE.

[48] Booth JE, Gaston KJ, Evans KL, Armsworth PR (2011). The value of species rarity in biodiversity recreation: A birdwatching example. Biol. Cons..

[49] Rowley JJ (2019). FrogID: Citizen scientists provide validated biodiversity data on frogs of australia. Herpetol. Conserv. Biol..

[50] Boakes EH (2016). Patterns of contribution to citizen science biodiversity projects increase understanding of volunteers recording behaviour. Sci. Rep..

[51] Garrard GE, McCarthy MA, Williams NS, Bekessy SA, Wintle BA (2013). A general model of detectability using species traits. Methods Ecol. Evol..

[52] Denis T (2017). Biological traits, rather than environment, shape detection curves of large vertebrates in neotropical rainforests. Ecol. Appl..

[53] Sólymos P, Matsuoka SM, Stralberg D, Barker NK, Bayne EM (2018). Phylogeny and species traits predict bird detectability. Ecography.

[54] Wood C, Sullivan B, Iliff M, Fink D, Kelling S (2011). eBird: Engaging birders in science and conservation. PLoS Biol.

[55] GBIF.org (3rd December 2019). *GBIF occurrence download*. 10.15468/dl.lpwczr

[56] Gilfedder M (2019). Brokering trust in citizen science. Soc. Nat. Resour..

[57] Callaghan C, Lyons M, Martin J, Major R, Kingsford R (2017). Assessing the reliability of avian biodiversity measures of urban greenspaces using eBird citizen science data. Avian Conserv. Ecol..

[58] Johnston, A. *et al.* Best practices for making reliable inferences from citizen science data: Case study using eBird to estimate species distributions. *BioRxiv* 574392 (2019).

[59] Myhrvold NP (2015). An amniote life-history database to perform comparative analyses with birds, mammals, and reptiles: Ecological archives E096–269. Ecology.

[60] Dale J, Dey CJ, Delhey K, Kempenaers B, Valcu M (2015). The effects of life history and sexual selection on male and female plumage colouration. Nature.

[61] R Core Team. *R: A Language and Environment for Statistical Computing* (R Foundation for Statistical Computing, 2020).

[62] Wickham H (2019). Welcome to the tidyverse. J. Open Source Softw..

[63] Bates D, Mächler M, Bolker B, Walker S (2015). Fitting linear mixed-effects models using lme4. J. Stat. Softw..

[64] Kuznetsova A, Brockhoff PB, Christensen RHB (2017). lmerTest package: Tests in linear mixed effects models. J. Stat. Softw..

[65] Johnston A (2014). Species traits explain variation in detectability of UK birds. Bird Study.

[66] Steen VA, Elphick CS, Tingley MW (2019). An evaluation of stringent filtering to improve species distribution models from citizen science data. Divers. Distrib..

[67] Henckel L, Bradter U, Jönsson M, Isaac NJ, Snäll T (2020). Assessing the usefulness of citizen science data for habitat suitability modelling: Opportunistic reporting versus sampling based on a systematic protocol. Divers. Distrib..

[68] Caley P, Welvaert M, Barry SC (2020). Crowd surveillance: Estimating citizen science reporting probabilities for insects of biosecurity concern. J. Pest. Sci..

[69] Périquet S, Roxburgh L, le Roux A, Collinson WJ (2018). Testing the value of citizen science for roadkill studies: A case study from South Africa. Front. Ecol. Evol..

[70] Nakagawa S, Freckleton RP (2011). Model averaging, missing data and multiple imputation: A case study for behavioural ecology. Behav. Ecol. Sociobiol..

[71] Schlossberg S, Chase M, Griffin C (2018). Using species traits to predict detectability of animals on aerial surveys. Ecol. Appl..

[72] Aristeidou M, Scanlon E, Sharples M (2017). Profiles of engagement in online communities of citizen science participation. Comput. Hum. Behav..

[73] Troscianko J, Skelhorn J, Stevens M (2017). Quantifying camouflage: How to predict detectability from appearance. BMC Evol. Biol..

[74] Schuetz JG, Johnston A (2019). Characterizing the cultural niches of North American birds. Proc. Natl. Acad. Sci..

[75] Lišková S, Frynta D (2013). What determines bird beauty in human eyes?. Anthrozoös.

[76] Tulloch AI, Possingham HP, Joseph LN, Szabo J, Martin TG (2013). Realising the full potential of citizen science monitoring programs. Biol. Cons..

[77] Kobori H (2016). Citizen science: A new approach to advance ecology, education, and conservation. Ecol. Res..

[78] Callaghan CT, Poore AG, Major RE, Rowley JJ, Cornwell WK (2019). Optimizing future biodiversity sampling by citizen scientists. Proc. R. Soc. B.

[79] Pacifici K (2017). Integrating multiple data sources in species distribution modeling: A framework for data fusion. Ecology.

[80] Robinson OJ (2020). Integrating citizen science data with expert surveys increases accuracy and spatial extent of species distribution models. Divers. Distrib..

[81] van Strien AJ, Termaat T, Groenendijk D, Mensing V, Kery M (2010). Site-occupancy models may offer new opportunities for dragonfly monitoring based on daily species lists. Basic Appl. Ecol..

[82] Van der Wal R (2015). Mapping species distributions: A comparison of skilled naturalist and lay citizen science recording. Ambio.

[83] Dennis EB, Morgan BJ, Brereton TM, Roy DB, Fox R (2017). Using citizen science butterfly counts to predict species population trends. Conserv. Biol..

[84] Stoudt, S., Goldstein, B. R. & De Valpine, P. Identifying charismatic bird species and traits with community science data. bioRxiv. 10.1101/2021.06.05.446577 10.1073/pnas.2110156119 PMC9169790 35412904

