# Supplementary material for: Large-bodied birds are over-represented in unstructured citizen science data
Source: Sci Rep. 2021 Sep 24;11:19073. doi: 10.1038/s41598-021-98584-7 (PMC8463711; doi:10.1038/s41598-021-98584-7)
Supplement: Supplementary file 1 — Supplementary Information 1. [file 41598_2021_98584_MOESM1_ESM.docx]

SUPPLEMENTARY FIGURES


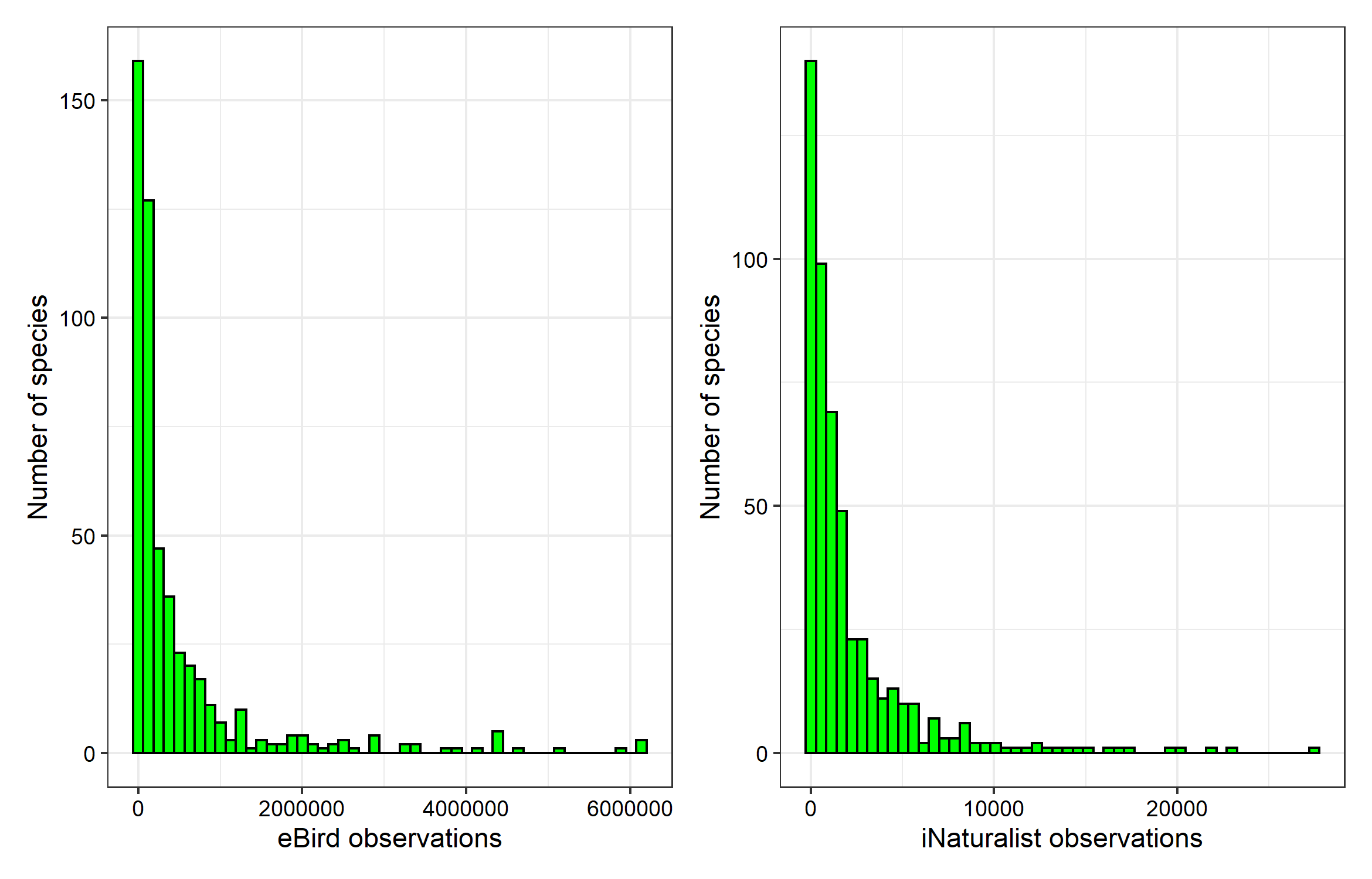
**Figure S1**. Histograms of the number of observations for a species from both eBird and iNaturalist citizen science projects.


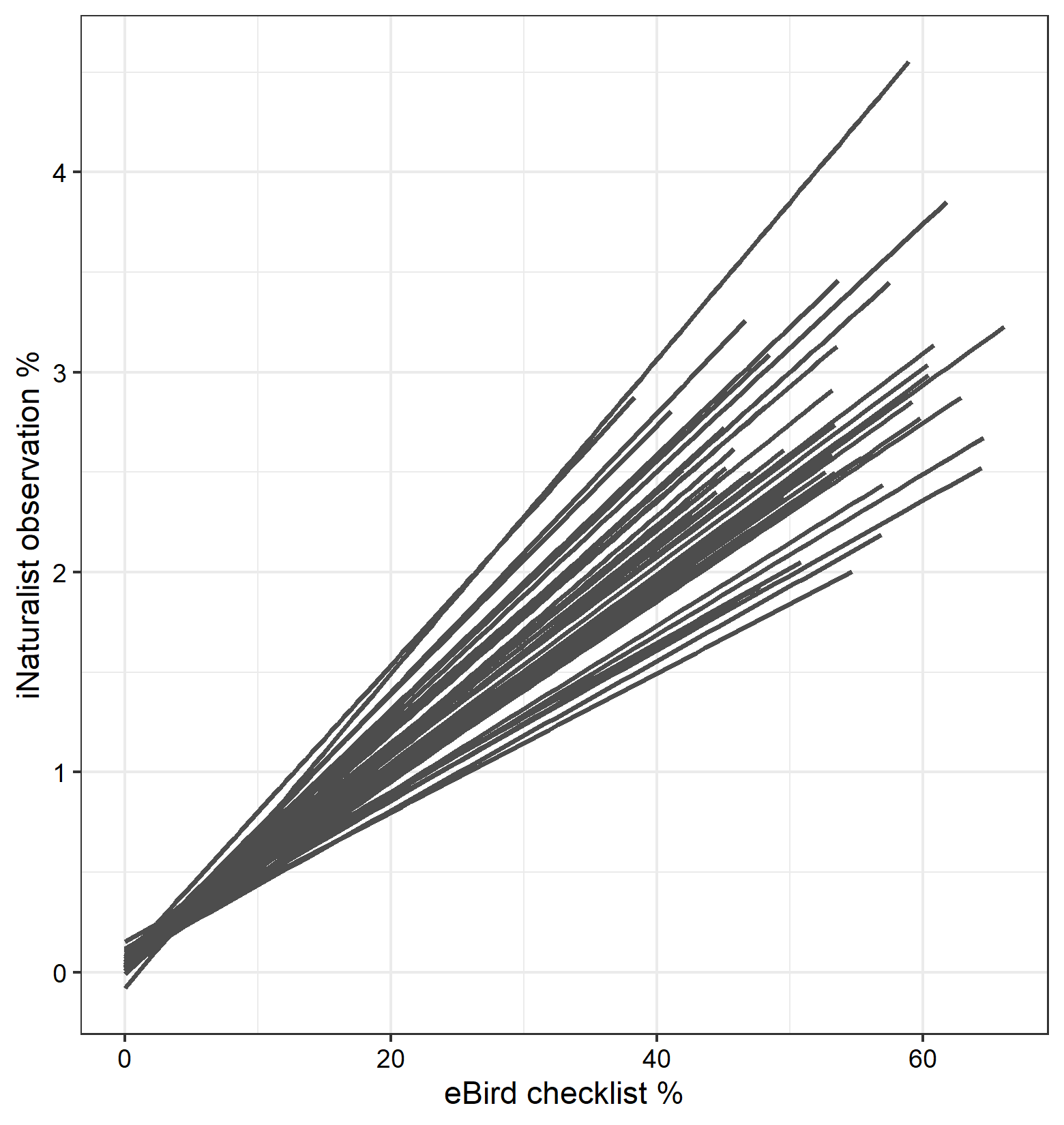


**Figure S2**. Among states (each line represents a state; N=49) we found that the percent of eBird checklists a species was found on and the percent of all iNaturalist observations a species comprised was strongly correlated.

**Table S1.** Uploaded separately. Raw data used for modelling, including the residual difference between iNaturalist and eBird, stratified by state.

**Table S2**. Results of single regression models, where each trait was treated separately, and consequently had different sample sizes in the model fit. Each model was fit with the residuals used as the response variable, the specific trait as the predictor variable, where body size and flock size were log10-transformed and IUCN was treated as an ordinal variable, and state was a random effect. This analysis was performed to confirm the results of the multiple regression mixed effects analysis presented in the main results (Figure 4).

|  | estimate | t | p-value | Number of obs |  | Number of species |
| --- | --- | --- | --- | --- | --- | --- |
| Body size | 0.11 | 31.59 | <0.001 | 7743 |  | 450 |
| Color | -0.01 | -0.413 | 0.68 | 4542 |  | 258 |
| Flock size | 0.033 | 6.118 | <0.001 | 8702 |  | 507 |
| IUCN status | 0.078 | 7.73 | <0.001 | 7629 |  | 442 |
|  |  |  |  |  |  |  |
